# The use and helpfulness of self-management strategies for depression: The experiences of patients

**DOI:** 10.1371/journal.pone.0206262

**Published:** 2018-10-25

**Authors:** Rosa A. van Grieken, Mirjam J. van Tricht, Maarten W. J. Koeter, Wim van den Brink, Aart H. Schene

**Affiliations:** 1 Department of Psychiatry, Academic Medical Center, University of Amsterdam, Amsterdam, The Netherlands; 2 Department of Psychiatry, Radboud University Medical Center, Nijmegen, The Netherlands; 3 Donders Institute for Brain, Cognition and Behavior, Radboud University, Nijmegen, the Netherlands; USC Keck School of Medicine, Institute for Global Health, UNITED STATES

## Abstract

**Objective:**

To explore which of 50 self-management strategies are actually used and which are perceived as most helpful by patients in their day-to-day management of depression, in order to empower patients and promote active engagement in their own care.

**Methods:**

Retrospective study using an online self-report survey to assess the use and perceived helpfulness of 50 previously identified self-management strategies in 193 participants who recently recovered from a major depressive episode.

**Results:**

Forty-five of the 50 strategies were used by at least half of all participants and about one third of all participants perceived almost 50% of all strategies as (very) helpful. The most used strategies, such as ‘finding strategies to create pleasurable distractions’, ‘engaging in leisure activities’ or ‘identifying the cause of the depression’, were not always perceived as most helpful. In addition, the perceived most helpful strategies, such as ‘completing treatment’ and ‘leaving the house regularly’ were not always the most used ones.

**Conclusions:**

Patients use and perceive a wide range of self-management strategies as helpful to recover from their depression. Patients use and perceive strategies about engagement in treatment and physical activities as being most helpful. These finding may contribute to the further development and implementation of self-management programs for the prevention or the rehabilitation of depression.

## Introduction

Depression is one of the most prevalent mental disorders, responsible for an impressive burden in terms of personal suffering, consequences for relatives and societal costs [1]. Patients, family members, mental health professionals and policy makers are pursuing (cost) effective ways to facilitate recovery, improve functioning and diminish health care burden [2,3]. One of the more recent possibilities to do so is the introduction of self-management strategies, which increasingly becomes an important priority in today’s approach to improve mental health [4–7]. Self-management entails the patient to be the expert of his or her own process of health improvement and recovery, including symptom management, dealing with the psychosocial consequences and lifestyle changes inherent to living with a chronic (relapsing) disease [8].

Self-management and enhancement of self-management strategies have been applied and studied for quite some time in the context of somatic chronic diseases [9–12]. Although mental health care has a strong tradition in conceptualizing chronicity, recovery and patient empowerment, self-management is less well established clinically and studies on its use and effectiveness are lacking behind. However interest is certainly growing [13]. Recently, Houle *et al*. (2013) [14] performed a systematic review in order to describe different self-management approaches for depression and to examine their efficacy. They concluded that the application of self-management seems to be associated with reduced depressive symptoms and improved functioning. Villagi *et al*. (2015) [15] identified 60 self-management strategies for patients with mood and anxiety disorders, ranging from breaking isolation and maintaining social relationships, implementing strategies to instil hope for recovery, engaging in sports activities and seeking professional help.

The different conceptualizations of self-management and the different techniques and approaches (e.g. e-health, booklets, (peer-led) group interventions with or without professional support) hinder and complicate research on the effectiveness of these strategies and consequently also hamper solid conclusions in reviews and meta-analyses [11,16,17]. Although results are promising, e.g. in achieving clinically meaningful improvements in depression outcomes, more robust evaluation is required in order to determine the effectiveness of self-management interventions. The heterogeneity of self-management may finally result in a situation where different parties, such as clinicians, health institutions and policy makers will be using the concept self-management according to their own beliefs and benefits about patient involvement and good chronic care.

When we started our research in self-management, the majority of self-management approaches were designed, implemented and led by mental health professionals. By definition self-management involves active participation of the patients themselves [18] and so we designed our projects from the principle to actively engage patients both in defining and conceptualizing self-management strategies and perspectives [18–19] and in quantifying which self-management strategies they consider to be useful and helpful in their recovery [20, 21]. In one of our previous studies we first identified 50 self-management strategies considered as helpful according to patients who recently had recovered from a depressive episode [22]. As a next step we developed for the current study a self-report survey to address the following research questions: 1) Which self-management strategies are used the most by patients with depression; and 2) Which strategies are perceived as most helpful?

## Methods

### Participants

We included participants who recently recovered from a depressive episode to ensure a vivid recollection of self-management strategies that they had used during their (last) illness period. Participants had to meet the following inclusion criteria: (1) 18 years or older; (2) recovered from a major depressive episode (MDE) within the last 3 years, (3) diagnosed by a professional; and (4) sufficient command of the Dutch language. Participants were excluded based on the following criteria: (1) clinically diagnosed with bipolar disorder; (2) incomplete or missing data regarding age or gender; and (3) score below 5 (no depression) on the *Patient Health Questionnaire-9* (PHQ-9) regarding their last depressive episode [23]. Bipolar disorder was assessed by a question to agree or disagree with ‘I do not have a bipolar or manic-depressive disease’. “Recovery” was assessed by a question ‘Have you been recovered from depression in the last 3 years?’

We aimed to recruit about 200 participants and used various methods: (a) we posted a request for study participation on several depression-related patient-websites; (b) we sent an e-mail request to the members of the Dutch patient organization “Depression Association”; and (c) we requested health professionals (psychologists, psychiatrists) working in various Dutch mental health institutions to invite their patients after treatment completion for study participation by giving an information letter. The information letter explained the objectives of the study, eligibility for study participation and provided a link to the secured, anonymous online survey platform.

The study was presented to the Medical Ethics Committee of the Academic Medical Center in Amsterdam. In line with the Dutch legislation, this Committee decided that the study did not require formal ethical review as participants were recruited on a volunteer basis and were not requested to undergo any potentially incriminating intervention and their participation was anonymous.

### Assessments

Using the 50 self-management strategies identified in our previous qualitative study with patients recently recovered from an episode of major depression [22], we developed an online survey (SurveyMonkey.com) to assess the use of self-management strategies and their perceived helpfulness. Prior to distribution of the survey, a pilot panel of experts, including patients (n = 13) and mental health professionals (n = 10), critically reviewed the items independently for their clarity and applicability. Based on their comments, the survey was adapted. For each of the 50 self-management strategies participants were first asked whether they had used that strategy during their most recent episode of depression (‘yes’ or ‘no’). If they did so, they were asked to indicate how helpful this strategy had been for the recovery of their depression on a 5-point Likert-type scale (1 = ‘not helpful at all’ and 5 = ‘very helpful’).

Depression severity during the most recent episode was assessed with the 9 questions of the PHQ-9 [23]. A 19-item questionnaire was developed to assess sociodemographic and clinical characteristics for subgroup comparisons.

### Data analysis

Data were analysed using the Statistical Package for the Social Sciences (IBM SPSS, version 21) for Windows [24]. Descriptive statistics were used to summarize the data such as sociodemographic and participant variables. For each self-management strategy, we first calculated the percentage of users and non-users. We then calculated the percentage of participants who used a certain strategy and perceived its use as ‘helpful’ or ‘very helpful’ (score 4 or 5).

## Results

### Participant characteristics

Of the 236 participants who started the survey, 193 (81.8%) met eligibility criteria. Of the 43 participants who were excluded, 23 had bipolar disorder, 11 had a PHQ-9 score < 5 during their last MDE, eight had an MDE more than three years ago and one had missing data on more than 20% of the strategies. Some participants met several exclusion criteria.

Demographic and clinical characteristics of the 193 eligible participants are presented in Table 1: 78% female, mean age 40 years (SD 13.5), 60% married/partner, 65% employed and 32% living alone. The mean PHQ-9 score during the last depressive episode was 18.68 (SD 4.98), indicating moderately severe to severe depression. 85% had two or more earlier episodes of depression, and 83% had a positive family history of depression.

**Table 1:** Demographic and clinical characteristics of participants (n = 193).

Demographic characteristics	N	(%)
Gender		
Male	43	(22.3)
Female	150	(77.7)
Age		
18–29 years	53	(27.5)
30–59 years	125	(64.8)
60+ years	15	(7.7)
Nationality		
Dutch	186	(96.3)
Surinam	3	(1.6)
Turkish	3	(1.6)
Moroccan	1	(0.5)
Relational status		
Single/separated	77	(39.9)
Married/partnership	116	(60.1)
Educational level		
Low/intermediate (primary/secondary school)	88	(45.6)
High (college or university)	105	(54.4)
Employment (%)		
Paid/voluntary work	125	(64.8)
Unemployed	68	(35.2)
Living condition		
Alone	61	(31.6)
With others (partner/children/family/friends)	132	(68.4)
Religion		
No	139	(72.0)
Yes	54	(28.0)
Clinical characteristics		
Family history with depression*		
None	34	(17.6)
First degree	130	(67.4)
Second/third degree	160	(82.9)
Age of onset of first depressive episode		
< 20 years	92	(47.7)
≥ 20 years	101	(52.3)
Lifetime number of depressive episodes		
Single episode	30	(15.5)
2–4 episodes	90	(46.6)
≥ 5 episodes	73	(37.8)
Lifetime number of depression treatments		
≤ 1	60	(31.1)
≥ 2	133	(68.9)
Duration of most recent depression		
< 8 weeks	38	(19.7)
9–32 weeks	65	(33.7)
> 32 weeks	90	(46.6)
Treating professional regarding most recent depression*		
No treatment	5	(2.6)
Family doctor	54	(28.0)
Psychologist	104	(53.9)
Psychiatrist	112	(58.0)
Patient Health Questionnaire (PHQ)-9 score		
Mild (score 5–14)	32	(16.6)
Moderate (score 15–19)	72	(37.3)
Severe (score 20–27)	89	(46.1)

* Including overlap (more options are possible).

### Most used strategies and their perceived helpfulness

**Fig 1:**
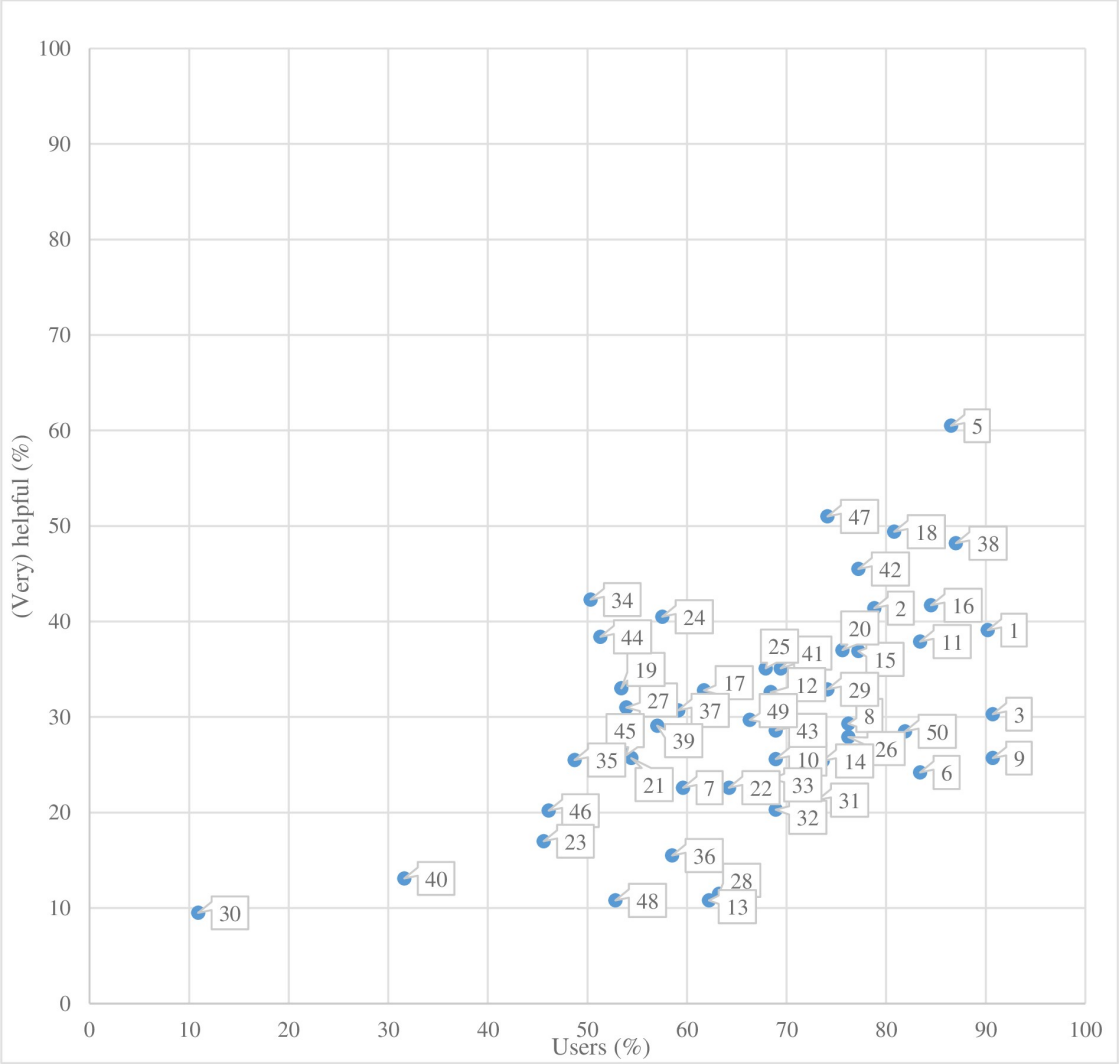
Relationship between the percentage of use and the perceived helpfulness of each of the 50 used self-management strategies.

With regard to the first research question (‘which self-management strategies are used most by patients with depression’), we found that 15 of the 50 strategies were used by more than 75% of the participants (by 76–91%), 30 strategies were used by 50–75% of the participants and only 5 of the 50 strategies were used by less than 50% of participants (see Fig 1). Thus, 45 of the 50 strategies were used by at least half of all participants.

With regard to the second question, we found that only one of the 50 strategies was perceived as (very) helpful by more than 60% of the participants (61%) and that only 15 strategies were considered as such by 35–60% of the participants. Most strategies, namely 35, were perceived as (very) helpful by less than 35% of the participants of which 5 were considered as such by less than 15% of the participants. Table 2 provides a detailed overview of all 50 strategies with corresponding percentages of use and perceived helpfulness.

**Table 2:** Fifty self-management strategies ordered according to the percentage of participants using a strategy.

Number	Strategy	Used		Very helpful (4 or 5)		
		N	(%)	(N)	(%)^1^	(%)^2^
1	Identifying the cause of the depression	174	90.2	68	39.1	35.3
2	Overcoming problems with concentration by creating to-do lists	152	78.8	63	41.4	32.6
3	Finding strategies to create pleasurable distractions	175	90.7	53	30.3	27.5
4	Finding information about depression	164	85	47	28.7	24.4
5	Completing treatment	167	86.5	101	60.5	52.3
6	Explaining depression to friends and family	161	83.4	39	24.2	20.2
7	Discussing changes in role within the family/relationship	115	59.6	26	22.6	13.5
8	Meeting up with friends regularly	147	76.2	43	29.3	22.3
9	Engaging in leisure activities	175	90.7	45	25.7	23.3
10	Explaining depression to manager	133	68.9	34	25.6	17.6
11	Engaging in moderate physical activity (cycling, walking etc.)	161	83.4	61	37.9	31.6
12	Creating a timetable of activities	132	68.4	54	32.6	14
13	Explaining depression to colleagues	120	62.2	13	10.8	7.6
14	Taking every opportunity to tidy the house	142	73.6	36	25.4	18.7
15	Setting realistic short term goals	149	77.2	55	36.9	28.5
16	Making sure you have a good day/night rhythm	163	84.5	68	41.7	35.2
17	Engaging in a structured form of meditation (e.g. yoga, mindfulness)	119	61.7	39	32.8	20.2
18	Ensuring enough rest to avoid exhaustion through over-exertion	156	80.8	77	49.4	39.9
19	Seeking contact with fellow sufferers	103	53.4	34	33	17.6
20	Engaging in sports activities	146	75.6	54	37	20
21	Keeping a diary	105	54.4	27	25.7	14
22	Observe alcohol intake	124	64.2	28	22.6	14.5
23	Being able to explain depression yourself	88	45.6	15	17	7.6
24	Finding a different therapist when there is limited progress	111	57.5	45	40.5	23.3
25	Finding meaningful occupations (e.g. volunteering)	131	67.9	46	35.1	23.8
26	Becoming aware of daily routines	147	76.2	41	27.9	21.3
27	Adjusting the discussion about depression allowing for what the partner/friend can cope with	104	53.9	32	31	16.7
28	Ignoring the tiredness associated with depression	122	63.2	14	11.5	7.3
29	Discussing depression with those you trust in order to have support nearby	143	74.1	47	32.9	24.4
30	Writing a web blog	21	10.9	2	9.5	1.3
31	Explaining depression to partner/family	141	73.1	30	21.3	15.6
32	Making plans for the future	133	68.9	27	20.3	14
33	Changing the negative aspect of daily routines	132	68.4	31	23.5	16.1
34	Finding someone new when the relationship between therapist and patient is not compatible	97	50.3	41	42.3	21.3
35	Asking for support at work	94	48.7	24	25.5	12.4
36	Recalling positive memories	113	58.5	18	15.5	9.1
37	Gradually resuming responsibilities that had been taken over by others	114	59.1	35	30.7	18.1
38	Leaving the house regularly	168	87	81	48.2	41.9
39	Exploring new hobbies	110	57	32	29.1	16.6
40	Restricting the time spent on worrying	61	31.6	8	13.1	4.1
41	Finding out which activities are achievable	134	69.4	47	35.1	24.4
42	Acknowledging that depression is a disease	149	77.2	68	45.5	35.1
43	Using a positive mantra	133	68.9	38	28.6	19.7
44	Organizing that a therapist is accessible	99	51.3	38	38.4	19.7
45	Searching out your family background	104	53.9	27	26	14
46	Discussing information found about depression with therapist	89	46.1	18	20.2	9.3
47	Making sure there is adequate support when using medication	143	74.1	73	51	37.8
48	Meeting up with people who are not aware of the depression	102	52.8	11	10.8	5.7
49	Including partner/family in the treatment	128	66.3	38	29.7	19.7
50	Healthy eating	158	81.9	45	28.5	23.3

^1^% of participants who have used the strategy

^2^% of all 193 participants

The relationship between the percentage of strategies used and the percentage perceived as (very) helpful is also shown in Fig 1. The figure indicates that the most used strategies are generally the most helpful ones. However, the figure also shows that the most used strategies (strategy 3 ‘finding strategies to create pleasurable distractions’, strategy 9 ‘engaging in leisure activities’, or strategy 1 ‘identifying the cause of the depression’) were not always perceived as most helpful. Also, the strategies perceived as most helpful (strategy 5 ‘completing treatment’, strategy 38 ‘leaving the house regularly’ or strategy 2 ‘overcoming problems with concentration by creating to-do lists’) are not always the most used ones. Reflecting on those upper right—most used and perceived most helpful—strategies, one could not unambiguously identify a common theme, although (physical) activity related strategies are well represented. The lower left strategies also show a clear correlation: The least used strategies are perceived the least helpful ones. Only 21 participants (11%) have used strategy 30 ‘writing a weblog’, with only 10% appreciating the strategy. Similarly, only 61 participants (32%) used strategy 40 ‘restricting the time spent on worrying’, and only 13% perceived the strategy to be very helpful. Participants seemingly explore and use all kinds of strategies, with a risk of not being helpful.

## Discussion

This study explored whether the 50 self-management strategies previously identified by patients for their recovery from depression, were actually used by them and which strategies were perceived as being most helpful. To the best of our knowledge, this is the first study evaluating the subjective effectiveness of these strategies by almost 200 participants of whom most had suffered from severe and recurrent depression.

Interestingly, although ‘avoidance behaviour’ as a reflection of the apathy and anhedonia that are core symptoms of depressive illness [25], patients mainly used and experienced other strategies as most helpful, e.g. strategies that focus on defining depression (e.g. ‘acknowledging that depression is a disease’, ‘identifying the cause of the depression’) and engaging in physical activities (e.g. ‘leaving the house regularly’, ‘engaging in moderate physical activities’, ‘engaging in sports activities). These results support—from the patient’s point of view—intervention studies that show the efficacy of self-management strategies as exercise or physical activity to reduce depression symptoms [26]. Furthermore, while the survey investigated particularly self-management strategies to be useful in daily life coping with depression, participants perceived ‘completing treatment’, ‘organizing that a therapist is accessible’ and ‘finding a different therapist when there is limited progress’ to be among the most useful strategies. Patients apparently perceive an active and critical attitude towards professional treatment as part of self-management, which indicates that professional support is not only accepted, but is also a critical part of their personal struggle with depression. So, one may speculate that self-management must be supported in alliance with a professional framework.

Of course we were interested to summarize the 50 strategies in a number of overarching self management themes. However, an exploratory factor analysis was not successful and we decided to exclude the results from this report. First, 17 of the 50 strategies had to be excluded, due to low factor loadings (<0,4). Secondly, the four identified factors together explained only 32% of the variance and third, the themes appeared not distinctive enough to help us in better understanding the concept self-management.

A question that arises is how exclusive are self-management strategies for depression only? Because, our set of strategies shows overlap with those that have been found in other studies of (minor to chronic) depression [19,21,27], but also with those for other mental disorders such as anxiety and bipolar disorders [5,15]. More interestingly we also found quite some overlap with self-management strategies for chronic somatic diseases like asthma or diabetes [8,28]. These self-management strategies mostly include changing life habits, behavioural activation and improving communication with the professional, family and friends. These results from different studies suggest that the same basic abilities are needed to efficiently manage one’s disease, regardless of it being a mental or somatic disease.

To create a simple overview for patients and professionals to use self-management strategies in clinical practice, it would be helpful if they were united into a core generic set of strategies for all chronic diseases [29]. An addendum with some unique strategies for the specific diseases may be necessary, such as ‘making sure you have a good day-night rhythm’ for depression. Especially for depression, a generic approach may help diminish the stigma [30], due to the realisation that depression is a disease like any other.

Despite today’s interest in self-management in mental health care, self-management for chronically mental ill patients remains relatively underdeveloped in Europe [31]. Furthermore, the various professional guidelines for depression hardly pay attention to self-management as a new approach in the treatment of depression [32–34]. For example, the only sentence in the depression guideline of the American Psychiatric Association [32] is about cases of incomplete recovery: ‘the professional should add a disease management component to the overall treatment plan … such as developing self-management skills’ (p.53). The results from our study add to the understanding of which strategies are actually used and perceived as helpful and may contribute to the development of new guidelines.

This research has been a first step in exploring the actual use and helpfulness of self-management strategies. However, since we measured the ‘patients’ perspective’, we cannot say more than those are the results from ‘their’ perspectives whether a participant did or did not recently used a particular strategy. The number of strategies was based on a foregoing qualitative study in which we explored the experiences of patients who recently recovered from a depressive episode about their own contribution to recovery during brainstorm sessions. The brainstorms resulted in 50 strategies which were included without selection in our questionnaire.

The perspectives of people are obviously subjective, and there is still a question for future research to find out e.g. to what extent the patients really used the strategies that they mentioned in an accurate way. An idea for the future could me to develop a structured depression self-management programme with the help of the results of this survey. The effectiveness of such a programme should be tested in a randomized clinical trial with a sufficient number of participants in order to determine whether the perceived helpful self-management strategies actually help in clinical practice. Next, no simple package or recipe suits every patient. Therefore, a personalized strategy selection may be needed for every patient that should be made in close collaboration of the patient and the professional.

### Strengths and limitations

Our study has several strengths. First, the use of qualitative data of the patient’s perspective in the development of a survey to obtain more quantitative data about the actual use and perceived helpfulness of strategies guarantees the ecological validity of the survey. Second, although this was a first exploration, use of a survey allowed us to examine self-management strategies in a relatively large and clinically diverse sample.

Of course the study also has limitations. A first limitation concerns the cross-sectional nature of the study, which makes it hard to estimate the actual influence of self-management on recovery. Prospective research is needed to better understand the association between the use of self-management strategies and clinical outcomes, while considering the stage or phase of the depression. Second, all measures relied on retrospective self-report and no data from medical records were available. Third, limiting the sample to those who have recovered from the depressive episode reduces the generalizability of the findings. We consciously discriminated this ‘recovered’ sample from a ‘currently depressed’ sample because strategies they use to recover or to cope with depression differ (van Grieken et al 2014). The sample was characterized by more severe and recurrent episodes of depression, which also means more ‘experienced’ patients. This selection might imply that our sample is a selection of patients who consider themselves as successful in self-management. Fourth, another limitation is that our sample was a culturally and ethnic homogeneous sample, which also did not include adolescents or older people. Future research might focus on differences between subgroups according to their use of self-management strategies.

### Conclusion

It can be concluded that there are a substantial number of self-management strategies that patients can use and perceive as helpful to recover from their depression. Patients used and perceived strategies about engagement in treatment and physical activities as being most helpful. This set of self-management strategies may be a valuable support to the current use of professional psycho-education and behavioural treatments for depression to empower patients and promote active engagement in their own care. We expect that patients are more motivated to use the strategies that promote engaging in activities instead of avoidance behavior with the knowledge that other patients perceived them as very helpful during their recovery.

### Practice implications

The most used strategies were not automatically perceived as the most helpful. We hypothese that each patient possibly uses a unique personalised mix of strategies, that suits his or her particular needs and interests, and that may change according to circumstances and over time. Therefore, we think that a focus only on the top 10 most used or perceived most helpful strategies would neglect the many other strategies that also were perceived helpful. We suppose a personalized selection is needed and the use of shared decision making as the preferred selection process [26].

This wide range of self-management strategies may help individual patients, self-help groups, carers and professionals to expand their treatment options. Professionals may discuss the strategies with their patients and carers during treatment and emphasize those strategies perceived as most helpful by other patients. Furthermore, these findings may contribute to the further development of a comprehensive self-management tool and to the implementation of self-management programs for the prevention or the rehabilitation of mild to severe, recurrent or chronic depression. Future research is needed to investigate the effectiveness of the strategies and to explore *how* the strategies could be introduced and supported in day-to-day life.
