# Correction: The use and helpfulness of self-management strategies for depression: The experiences of patients

**DOI:** 10.1371/journal.pone.0209109

**Published:** 2018-12-13

**Authors:** Rosa A. van Grieken, Mirjam J. van Tricht, Maarten W. J. Koeter, Wim van den Brink, Aart H. Schene

The title of Table 2 is incorrect. Please see the corrected Table 2 here.

**Table 2:** Fifty self-management strategies with corresponding percentages of use and perceived helpfulness.

Number	Strategy	Used		Very helpful (4 or 5)		
		N	(%)	(N)	(%)^1^	(%)^2^
1	Identifying the cause of the depression	174	90.2	68	39.1	35.3
2	Overcoming problems with concentration by creating to-do lists	152	78.8	63	41.4	32.6
3	Finding strategies to create pleasurable distractions	175	90.7	53	30.3	27.5
4	Finding information about depression	164	85	47	28.7	24.4
5	Completing treatment	167	86.5	101	60.5	52.3
6	Explaining depression to friends and family	161	83.4	39	24.2	20.2
7	Discussing changes in role within the family/relationship	115	59.6	26	22.6	13.5
8	Meeting up with friends regularly	147	76.2	43	29.3	22.3
9	Engaging in leisure activities	175	90.7	45	25.7	23.3
10	Explaining depression to manager	133	68.9	34	25.6	17.6
11	Engaging in moderate physical activity (cycling, walking etc.)	161	83.4	61	37.9	31.6
12	Creating a timetable of activities	132	68.4	54	32.6	14
13	Explaining depression to colleagues	120	62.2	13	10.8	7.6
14	Taking every opportunity to tidy the house	142	73.6	36	25.4	18.7
15	Setting realistic short term goals	149	77.2	55	36.9	28.5
16	Making sure you have a good day/night rhythm	163	84.5	68	41.7	35.2
17	Engaging in a structured form of meditation (e.g. yoga, mindfulness)	119	61.7	39	32.8	20.2
18	Ensuring enough rest to avoid exhaustion through over-exertion	156	80.8	77	49.4	39.9
19	Seeking contact with fellow sufferers	103	53.4	34	33	17.6
20	Engaging in sports activities	146	75.6	54	37	20
21	Keeping a diary	105	54.4	27	25.7	14
22	Observe alcohol intake	124	64.2	28	22.6	14.5
23	Being able to explain depression yourself	88	45.6	15	17	7.6
24	Finding a different therapist when there is limited progress	111	57.5	45	40.5	23.3
25	Finding meaningful occupations (e.g. volunteering)	131	67.9	46	35.1	23.8
26	Becoming aware of daily routines	147	76.2	41	27.9	21.3
27	Adjusting the discussion about depression allowing for what the partner/friend can cope with	104	53.9	32	31	16.7
28	Ignoring the tiredness associated with depression	122	63.2	14	11.5	7.3
29	Discussing depression with those you trust in order to have support nearby	143	74.1	47	32.9	24.4
30	Writing a web blog	21	10.9	2	9.5	1.3
31	Explaining depression to partner/family	141	73.1	30	21.3	15.6
32	Making plans for the future	133	68.9	27	20.3	14
33	Changing the negative aspect of daily routines	132	68.4	31	23.5	16.1
34	Finding someone new when the relationship between therapist and patient is not compatible	97	50.3	41	42.3	21.3
35	Asking for support at work	94	48.7	24	25.5	12.4
36	Recalling positive memories	113	58.5	18	15.5	9.1
37	Gradually resuming responsibilities that had been taken over by others	114	59.1	35	30.7	18.1
38	Leaving the house regularly	168	87	81	48.2	41.9
39	Exploring new hobbies	110	57	32	29.1	16.6
40	Restricting the time spent on worrying	61	31.6	8	13.1	4.1
41	Finding out which activities are achievable	134	69.4	47	35.1	24.4
42	Acknowledging that depression is a disease	149	77.2	68	45.5	35.1
43	Using a positive mantra	133	68.9	38	28.6	19.7
44	Organizing that a therapist is accessible	99	51.3	38	38.4	19.7
45	Searching out your family background	104	53.9	27	26	14
46	Discussing information found about depression with therapist	89	46.1	18	20.2	9.3
47	Making sure there is adequate support when using medication	143	74.1	73	51	37.8
48	Meeting up with people who are not aware of the depression	102	52.8	11	10.8	5.7
49	Including partner/family in the treatment	128	66.3	38	29.7	19.7
50	Healthy eating	158	81.9	45	28.5	23.3

^1^% of participants who have used the strategy

^2^% of all 193 participants
